# A Mid-Term Follow-Up in Patients with Symptomatic Moderate to Severe and Severe Degenerative Mitral Valve Regurgitation After Transapical NeoChord Implantation

**DOI:** 10.3390/biomedicines13071751

**Published:** 2025-07-17

**Authors:** Argyro Kalompatsou, Dimitris Tousoulis, Yannis Dimitroglou, Eirini Beneki, Panagiotis Theofilis, Konstantinos Tsioufis, Constantina Aggeli, Vasilis Lozos

**Affiliations:** 1 First Department of Cardiology, “Hippokration” General Hospital, National and Kapodistrian University of Athens, 11527 Athens, Greece; iro.kalompatsou@gmail.com (A.K.); dimiyann@hotmail.com (Y.D.); e.beneki@hotmail.com (E.B.); panos.theofilis@hotmail.com (P.T.); ktsioufis@gmail.com (K.T.); dina.aggeli@gmail.com (C.A.); lozosvasilis@yahoo.com (V.L.); 2 Department of Cardiology, Lausanne University Hospital and University of Lausanne, 1011 Lausanne, Switzerland

**Keywords:** mitral valve repair, NeoChord implantation, primary mitral valve regurgitation

## Abstract

**Background:** The transapical off-pump NeoChord procedure is a recognized minimally invasive surgical approach for the treatment of severe degenerative mitral regurgitation. This study aims to report the initial Greek experience with the NeoChord procedure, presenting mid-term clinical and echocardiographic outcomes from a single cardiothoracic surgical center, with a median follow-up duration of 20 months. **Methods:** In this study, 42 symptomatic patients with moderate to severe and severe primary mitral regurgitation underwent mitral valve repair with the Neochord procedure between March 2018 and December 2024. All patients were evaluated clinically and echocardiographically by the Heart team preoperatively, after 1 month, and at the last follow-up (end of 2024). The primary endpoint was established as the presence of a major clinical event (all-cause mortality, reintervention due to deterioration of MR, and cardiac-related rehospitalization). **Results:** The median age of patients was 69 [61.75–79.25] years, and 69% of patients were men. The median EuroScore II was 1.79 [1.32–2.48], and the STS-PROM MV repair score was 3.18 [2.28–4.66]. Regarding the preprocedural mitral valve anatomical evaluation, 35 patients had type A (83.3%),4 had type B(9.5%), whereas only two patients had type C and 1 with type D anatomy. The median of LAI was 1.2 [1.15–1.25], whereas the CI was 4 [2.15–5]. More than two neochordae were implanted in 34 patients (81%). MR severity improved at 1-month (<moderate:92.85%) and at the last follow-up (<moderate:92.1%). NYHA class decreased within 1 month (I + II: 95.23%) after the procedure and was maintained at the last follow-up (I + II: 94.73%). The median left ventricular ejection fraction (LVEF) before the procedure was 63 [58–67]%, which significantly decreased to 57 [53–61]% at the 1-month follow-up (2-sided *p* < 0.001). At the final follow-up, LVEF increased to 65 [60–68]%, however, this change was not statistically significant compared to the preprocedural value. During the follow-up period, four deaths were documented—three due to non-cardiac and one attributable to a cardiac cause. Two cases proceeded to reoperation for surgical valve implantation due to recurrent mitral valve regurgitation 6 months and 8 months after the NeoChord procedure. **Conclusions:** Transapical off-pump NeoChord implantation offers a minimally invasive alternative to conventional surgery for symptomatic patients with moderate-to-severe or severe primary mitral regurgitation. Among patients with suitable mitral valve anatomy, the procedure has demonstrated a favorable safety profile and promising mid-term outcomes, in terms of cardiac mortality, as well as freedom from reoperation and rehospitalization.

## 1. Introduction

Mitral regurgitation (MR) is the most common valve disease in the general population. It is divided into primary, which is characterized by lesions of one or more components of the mitral valve (MV) apparatus, or secondary, as a disease of the left ventricle or atrium resulting from an imbalance between closing and tethering forces. According to the European Society of Cardiology (ESC) guidelines [1], patients with severe primary MR should undergo mitral valvuloplasty by an experienced surgical team to keep the integrity of the MV anatomy as well as the subvalvular apparatus. Additionally, MV valvuloplasty should be the first option defined by the Heart Team in cases with high expectations for surgical success and preservation of the results. Mitral valve repair has arisen as the best treatment option for patients with primary MR, due to long-term outcomes, low risk of recurrence and reoperation, and low mortality rate [1,2].

Mitral valve repair mainly consists of annuloplasty, leaflet resection, and prosthetic chordae repair [3]. While surgical mitral valve repair is associated with favorable long-term outcomes, it necessitates thoracotomy and the use of extracorporeal circulation, rendering it a highly invasive intervention. Conversely, minimally invasive interventional repair techniques can be performed on a beating heart without the need for extracorporeal support, thereby reducing surgical trauma and facilitating a more rapid postoperative recovery. For patients with severe primary mitral regurgitation who are deemed ineligible for surgery or present with a high operative risk, transapical mitral valve neochord implantation under transesophageal guidance provides a safe and effective therapeutic alternative [1,4,5].

The NeoChord transapical off-pump MV repair is an established minimally invasive surgical procedure for reducing MR in patients with favorable MV anatomy [2,3,4,5,6,7,8,9]. The NeoChord procedure was performed using the NeoChord DS1000 Artificial Chordae Delivery System (NeoChord, Inc., St. Louis Park, MN, USA), guided by real-time two-dimensional and three-dimensional transesophageal echocardiographic imaging. By obtaining transapical access, three or more expanded polytetrafluoroethylene (ePTFE) chordae are positioned to the mitral valve. Notably, the accurate positioning, optimal neochordae length, and appropriate tensioning of the prolapsing leaflets are effectively achieved through three-dimensional transesophageal echocardiographic guidance [3,4,5,6,7].

The study aimed to present the first Greek experience of transapical NeoChord procedure, providing a mid-term follow-up in 20 [11.75–39] months in a single center of cardiothoracic surgery.

## 2. Materials and Methods

### 2.1. Patient Population

In the current study, 43 symptomatic patients with moderate to severe and severe degenerative mitral valve regurgitation, who underwent MV repair with the NeoChord procedure at the Hippokration General Hospital of Athens between March 2018 and December 2024, were enrolled. One patient was excluded from the study due to the occurrence of left atrial posterior wall dissection during the procedure. The Surgical Team decided to convert the initial surgical plan to MV replacement, and the procedure was completed successfully [10]. Exclusion criteria included the pleural disease, active endocarditis, coexistence of other valve disease, and unfavorable anatomy of the mitral valve, defined as extensive valve calcification or leaflet perforation [11,12,13,14].

### 2.2. Preoperative Evaluation

All patients underwent preoperative transthoracic echocardiography (TTE) and transesophageal echocardiography (TEE) to depict the anatomical data of the mitral valve apparatus. Moreover, the 3D-TEE offered more accurate information regarding the precise anatomy, the origin, and the direction of MR jet(s). The patients’ surgical risk was determined using the EuroScore II and the Society of Thoracic Surgeons Predicted Risk of Mortality Mitral Valve repair score (STS-PROM MV repairscore) for mitral valve repair [8,9]. Clinical assessment of patients’ symptomatic profile was evaluated with the New York Heart Association (NYHA) functional class grading system. All patients received the optimal guideline-directed medical therapy. The severity and mechanism of MR have been analyzed by TTE and TEE using Philips echo machine EPIQ 7 system (X5-1c, X8-2t probes) (Philips, Eindhoven, The Netherlands). The patient population was categorized based on the preprocedural 3D TEE study of MV morphology:

- Type A, isolated central posterior leaflet prolapse/flail;
- Type B, posterior multi-segment prolapse/flail;
- Type C, anterior or bileaflet prolapse/flail; and
- Type D, paracommissural prolapse/flail, or any disease with significant leaflet/annular calcifications [9].

The most important TEE echocardiographic parameter for patient selection is the leaflet-to-annulus index (LAI) ratio, which comprises the sum of posterior mitral leaflet (PML) and anterior mitral leaflet (AML) lengths divided by the antero-posterior diameter (AP) [(PML + AML):AP]. LAI measurements were obtained during mid-systole at the A2/P2 scallops level, using the mid-esophageal long-axis view. Patients with an LAI greater than 1.2 were considered to have sufficient leaflet tissue to achieve effective coaptation following the repair procedure [10,11,12,13,14,15,16]. An LAI value of ≥1.2 indicates that the combined leaflet length is approximately 20% greater than the AP. This redundant leaflet tissue serves as a potential coaptation surface, which can be effectively utilized following restoration of posterior leaflet mobility through the NeoChord repair procedure [12]. A value greater than 1.2 has been shown to be significantly correlated, maintaining favorable one-year outcomes [16]. Additionally, the coaptation index (CI) is defined as the ratio of the sum of PML and AML length minus AP divided by 2: [PML + AML-AP]:2 [11,12,13,14,15,16]. The coaptation length index is a reliable predictor of sufficient tissue overlap to attain a postoperative coaptation length ranging from 3 to 5 mm. CI value > 5 mm indicates optimal anatomy of the mitral valve and offers supplementary insight into the feasibility and potential success of the technique [14,15,16,17,18].

### 2.3. Technical Description of Neochord Implantation

**Figure 1:**
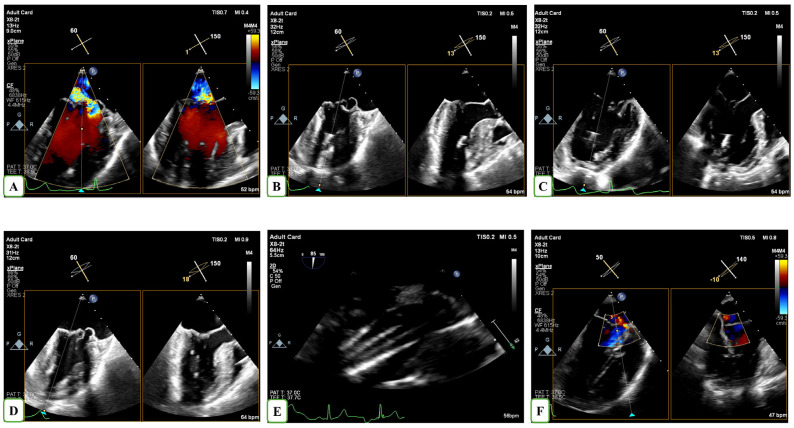
TEE-guided insertion of the NeoChord device through the left ventricular apex in a patient with severe MR (**A**). The TEE-guided procedure is illustrated in panels (**B**–**D**) using X-Plane modality to acquire the bicommisural and 3-chamber views. Four neochordae were implanted on the posterior leaflet, as visualized using the transgastric echocardiographic view (**E**). Color Doppler revealed residual trivial mitral regurgitation following NeoChord implantation (**F**).

The technical part of the procedure has been described in our previous publication [13]. MV repair was conducted under general anaesthesia via a left minithoracotomy approach, lateral to the apex. The NeoChord device was introduced into the left ventricle (LV) using 3D TEE guidance and the precise leaflet capture as well as the optimization of chordal length was performed appropriately (Figure 1). Procedural success was defined as the implantation of at least two neochordae and the reduction of MR to trivial-mild and mild to moderate at the end of the procedure.

### 2.4. Post-Procedural Follow-Up of the Study Population

All patients received guideline-directed medical therapy in accordance with the European Society of Cardiology guidelines. Clinical and echocardiographic assessments were performed at 1, 3, 6, and 12 months, and annually thereafter, either at Hippokration Hospital or at another institution based on patient preference. Both the initial and final follow-up visits were conducted in our department. The median follow-up for the study population was 20 [12–39] months. MR severity was evaluated during follow-up as trivial-mild, mild-moderate, moderate-severe, and severe. The primary endpoint was defined as the occurrence of major clinical events (all-cause mortality, reintervention due to deterioration of MR, and cardiac-related rehospitalizations).

### 2.5. Statistical Analysis

Data were analyzed using the SPSS software (SPSS, IBM SPSS Statistics, version 29.0.2.0, Armonk, NY, USA). Demographic and baseline variable data are reported as frequency (%),while continuous variables are summarized using medians and interquartile ranges (IQR, 25th to 75th percentiles). Comparisons of continuous variables were performed using the Wilcoxon signed-rank test. For all analyses, a two-sided *p* < 0.05 was statistically significant. We also conducted a post hoc power analysis, which showed that all statistically significant variables achieved a power greater than 0.95. The event-free survival curve was estimated using the Kaplan–Meier method.

### 2.6. Ethics

Written informed consent was obtained from all participants prior to their inclusion in the study. The research was conducted in full compliance with the ethical principles of the Declaration of Helsinki and followed all institutional guidelines to protect patient confidentiality and ensure data security. The study was approved by the ethical committee of Hippokration General Hospital of Athens (Protocol Code: 22532/19-12-2023, Approval Date: 21 December 2023). The authors declare no conflicts of interest. The study was not supported by any external funding sources that could have affected its design or analysis.

## 3. Results

The median age of patients was 69 [61.75–79.25] years, and 69% of patients were men. Demographic data were summarized in Table 1. Patient population surgical risk for MV replacement was defined as Euroscore II 1.79 [1.32–2.48] and STS-PROM MV repair score was 3.18 [2.28–4.66]. Eighteen patients had known coronary artery disease, whereas only one patient had had a previous MV valvuloplasty.

The preprocedural anatomical MV parameters are illustrated in Table 2. Most of the patients had type A (83.3%) and B anatomy (9.5%), whereas only two patients had type C and 1 with type D. More than two neochordae were implanted in the majority of patients (81%). The median of LAI was 1.2 [1.15–1.25], and the CI was 4 [2.15–5] mm.

**Figure 2:**
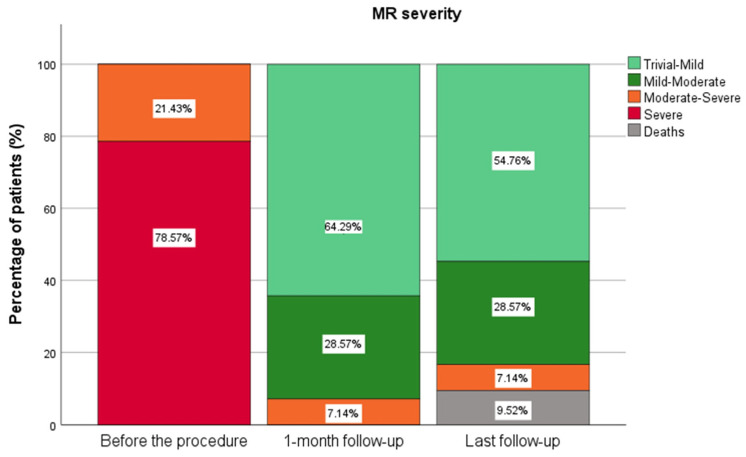
MR severity grade before the procedure, one-month follow-up, and the last follow-up.

In all cases, the grade of MR was successfully reduced postoperatively. All patients were discharged within two to three days following the procedure. Before the intervention, nine patients had moderate-to-severe mitral regurgitation (MR), while 33 patients presented with severe MR. At the one-month follow-up, 27 patients exhibited trivial-to-mild MR, 12 had mild-to-moderate MR, and three showed moderate to severe residual MR. Of the 42 patients, 38 were alive during the last follow-up. The favorable MR outcomes were sustained, with 23 patients showing trivial-to-mild MR, 12 exhibiting mild-to-moderate MR, and three presenting with moderate-to-severe residual MR (Figure 2).

**Figure 3:**
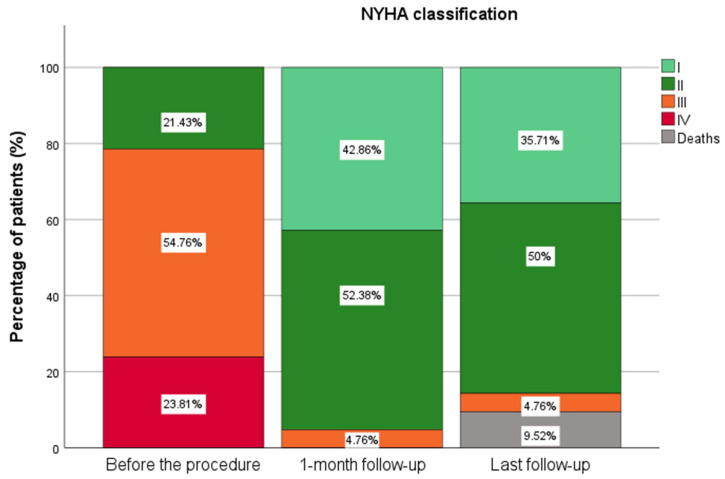
NYHA classification before the procedure, one-month follow-up, and the last follow-up.

**Figure 4:**
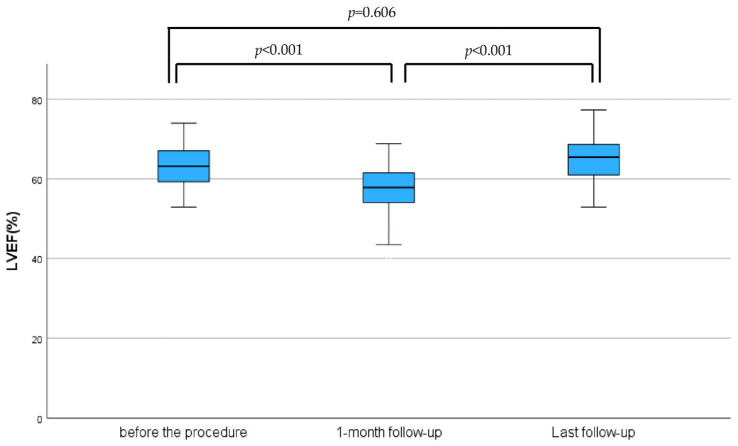
Medians of left ventricular ejection fraction (LVEF) before the procedure, one-month follow-up, and at last follow-up.

Similarly, NYHA class was improved immediately after the NeoChord implantation, and these results were preserved during mid-term follow-up. Before the procedure, nine patients were classified as NYHA class II, 23 as NYHA class III, and 10 as NYHA class IV. At the one-month follow-up, 18 patients were NYHA I, 22 were NYHA II, and two remained NYHA III. At the time of the final follow-up, 15 patients were classified as NYHA class I, 21 as class II, and two patients as class III (Figure 3). The median of left ventricular ejection fraction (LVEF) before the procedure was 63 [58–67]%, which significantly decreased to 57 [53–61]% at the one-month follow-up. At the final follow-up, LVEF increased significantly to 65 [60–68]%, however, this change was not statistically significant compared to the preprocedural (Figure 4).

**Figure 5:**
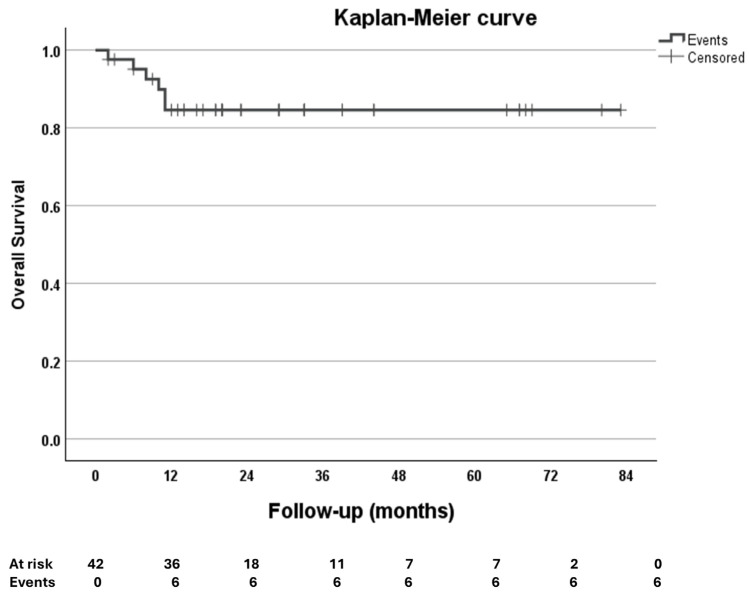
Kaplan–Meier event-free survival curve.

The overall survival and incidence of cardiovascular events were depicted using a Kaplan-Meier survival curve (Figure 5). From March 2018 to December 2024, four patient deaths were recorded, three of which were attributed to non-cardiac causes. Two 80-year-old men and one 82-year-old woman died 11 months and 10 months after the procedure, respectively. The causes of death were sepsis in the first two cases and an embolic event in the third. The cardiac death referred to a 69-year-old man with multiple comorbidities. Two months after the NeoChord procedure, the patient was diagnosed with MV endocarditis and died during the reoperation procedure for MV replacement.

Furthermore, during follow-up, two cases proceeded to reoperation, surgical valve implantation due to recurrent mitral valve regurgitation six months and eight months after the NeoChord implantation, respectively. The first patient had no ideal MV anatomy for the Neochord procedure. The LAI index was calculated as 1.1 (lower than 1.2), and the coaptation index was small (2 mm), whereas, posterior mitral annulus calcification was detected (type C). Moreover, the second one had complex MV anatomy of the posterior leaflet with P2a, P2b scallops, and an indentation between them. The anterior leaflet also demonstrated a pseudoprolapse part. This case was classified as a Type B candidate for the NeoChord treatment (P1-P2-P3 involvement). Due to the presence of multi-segmental disease, the LAI and coaptation index could not be reliably calculated. A newly ruptured chord originating from the anterior leaflet was identified during the subsequent surgical procedure. Despite the stability of the four previously implanted chords, the reoperative procedure necessitated the implantation of a prosthetic valve, tricuspid annuloplasty ring placement, and atrial fibrillation ablation. Interestingly, while the four implanted chords remained stable, the reoperation procedure included a prosthetic valve and tricuspid ring implantation as well as atrial fibrillation ablation.

## 4. Discussion

The follow-up data concerning the results of minimally invasive procedures such as transapical NeoChord implantation are limited. This study presented the first Greek experience of the mid-term follow-up of 42 symptomatic patients with moderate to severe and severe degenerative MR. While the first experience of transapical NeoChord implantation began in 2018, the enrollment of patients was significantly delayed during the COVID-19 pandemic. According to the current study results, elderly patients with moderate surgical risk successfully underwent the NeoChord procedure with favorable mid-term follow-up data. Even though the specific echocardiographic parameters in our population were not ideal as expressed by LAI and CI, the MR severity as well as the NYHA class improved significantly one month and at the last follow-up.

Furthermore, the LVEF decreased immediately after the procedure and improved during the most recent follow-up assessment. The initial decrease in LVEF is attributable to both a reduction in preload and an increase in afterload. The decrease of LVEF at the one-month follow-up and the increase of LVEF at the last follow-up were statistically significant.

According to the results of the current study, during follow-up, one cardiac related death was observed, two cases of rehospitalizations and three non-cardiac deaths occurred. As illustrated in Figure 4, all remaining patients experienced no major adverse events.

Interestingly, both patients who required surgical valve implantation for recurrent mitral regurgitation exhibited anatomically unfavorable mitral valve apparatus anatomy. In the first case, the anatomical criteria were suboptimal, with an LAI index of 1.1—below the ideal cutoff of 1.2—and a markedly reduced coaptation index. A short CI was predictive of suboptimal leaflet coaptation following the procedure. Additionally, the presence of posterior mitral annular calcification represented an adverse morphological parameter (type C anatomy). For the aforementioned reasons, the Heart Team initially recommended mitral valve replacement as the preferred treatment option, however, the patient declined. After thorough discussion, transapical NeoChord implantation was proposed as an alternative, albeit with limited expectations for a favorable outcome. The second patient had a complex MV anatomy, characterized by the presence of P2a and P2b scallops with an indentation. Moreover, the A2 and A3 segments appeared as pseudoprolapsed regions, resulting from inadequate support provided by the P2 and P3. Two distinct MR jets were observed: one central (a flail part of the posterior leaflet) and the other eccentric, directed. Although the patient was classified as a Type B candidate for the NeoChord procedure, multiple mitral valve segments were involved. As presented in the Section 3, the LAI and CI could not be reliably calculated due to the involvement of multiple segments (P2a, P2b, A2, and A3). The mitral valve was replaced with a new prosthetic valve, tricuspid ring implantation as well as atrial fibrillation ablation. It is worth emphasizing that clinical outcomes are determined not only by the type of MV but also by the complexity of the MV anatomy structure.

It is noteworthy that within the study population, a 61-year-old patient had previously undergone mitral valvuloplasty (MV ring and two chords implantation) and Percutaneous Coronary Intervention (PCI), two years and six months prior to the Neochords procedure, respectively. The new deterioration of MR was attributed to the rupture of previously implanted chordae. There is great interest in the Cardiothoracic Community concerning the role of transapical chord implantation in this specific population of patients with a previous mitral valvuloplasty as a Redo procedure. Three neochordae were implanted successfully, and the patient remained NYHA II class during the last follow-up.

It is worth highlighting that our initial experience, including immediate and short-term follow-up at six months, was previously published in 2020 in the Hell J Cardiol [13]. According to those initial results, patients who underwent transapical NeoChord implantation demonstrated excellent immediate and short-term (six-month) outcomes, with notable improvement in both NYHA functional class and mitral regurgitation severity

Colli et al. [8] presented a large series of patients who had received neochordae implantation and analyzed the possible mechanisms of recurrent MR. Among the key points responsible for recurrent regurgitation, patient selection (17.3%), technical issues (28.8%), progression of baseline disease (15.4%), left ventricle reverse remodeling (1.9%), excessive over-tensioning (35.8%), and PML curling (30.8%) were identified. According to those results, the main problem was the unfavorable mitral valve anatomy based on mitral annular calcification as well as the fragile leaflets, leading to possibly poorly calculated LAI. Furthermore, in published studies, cases of neochordae rupture were reported [14,15,16,17,18,19]. The potential causes are primarily attributed to prolonged friction between dissimilar materials (ePTFE, polypropylene, pledgets), excessive over-tensioning, and inadvertent neochordae damage during intraoperative handling. Nevertheless, robust data that definitively substantiate these assumptions remain unavailable at present.

Another critical intraoperative consideration was the degree of neochordal tension applied during the procedure. In accordance with the extensive experience of Colli et al. as well as our own clinical experience, the overtensioning of implanted neochordae represents a critical consideration that requires careful attention and expertise from the operating surgeon [11,12,13,14,15]. It is essential to take into account left ventricular remodeling during the follow-up period. In such cases, positive remodeling is expected to result in the relaxation of the neochordae. Conversely, the remodeling of the annulus, particularly in the absence of calcification, plays a crucial role [12,15,18,19,20,21,22,23].

The morphology of the mitral annulus and the extent of calcification are key determinants in the successful implantation of NeoChord. The mitral valve annulus is not rigid; therefore, positive left ventricular remodeling may also contribute to annular remodeling. Therefore, the initial theoretical limitation regarding the absence of a combined technique (involving both leaflets and annulus) may not be accurate. The positive LV remodeling and the flexible mitral ring should contribute to favorable MR results. Positive annular remodeling has been observed as a strong impact on patient heart function. However, the role of annular stabilization remains a topic of ongoing debate, as it has been proposed as a predictor of repair durability in conventional surgical procedures. In our view, this remains a significant concern, and further studies are required to validate the impact of NeoChord implantation on predicting favorable patient outcomes.

The first Canadian Clinical experience derived by a single Cardiothoracic Center [16]. Among 10 patients (mean age 76 years), the procedure was successfully completed in 9 patients, with an average of 3 chords implanted. The procedure was converted to an open surgery with mitral valve replacement. At the 6-week follow-up assessment, the majority of patients demonstrated either no or trivial MR, and two patients had moderate MR. It is noteworthy that the study population included two patients with a history of prior MV valvuloplasty.

Even with conventional surgical repair techniques, a certain proportion of patients experience recurrence. Recently, Kemin Liu et al. [21] published a study focusing on the outcomes following surgical repair of anterior, posterior, or bileaflet mitral valve prolapse. In a large cohort of 1192 patients with a mean age of 55 years, the majority (60.6%) presented with posterior leaflet prolapse. The 10-year cumulative survival and reoperation incidences among AML, PML, and bileaflet repairs were not significantly different. However, AML repair exhibited a higher cumulative incidence of recurrent mitral regurgitation compared to PML repair. These findings highlight the significance of leaflet involvement, particularly the AML, which is associated with a worse prognosis.

A novel ePTFE chordal implantation device, E-Chord (Med-Zenith Medical, Beijing, China), was recently developed in China. This study reported the initial results of this device in a porcine model [22]. Interestingly, the clinical evaluation should be very interesting as a different approach to mitral valve leaflets should be added. Smaller device that punctures the leaflets without insertion of the device (T shape) into the left atrium.

### Limitations

The results are based on a small cohort of consecutive patients managed in a single cardiothoracic center, limiting the generalizability of the study and the extrapolation of the findings to all patients with degenerative mitral regurgitation and suitable anatomical characteristics. The sample size limited the statistical power of the study, particularly with regard to detecting rare complications or performing reliable subgroup analyses. In addition, only the initial and final follow-up assessments were performed at our department, while all other evaluations were conducted at institutions chosen by the patients. Consequently, comprehensive annual echocardiographic follow-up data were not consistently available. On the other hand, a notable strength of this study is that the same experienced cardiothoracic surgical team, in conjunction with a dedicated cardiovascular imaging group specializing in this technique, established a coordinated and systematic approach during that period, which facilitated consistent interdisciplinary collaboration and longitudinal patient follow-up. The Heart Team thoroughly evaluated all clinical and anatomical data both prior to and during the procedure, and subsequently conducted follow-up for each patient. Given the anatomical variability among patients, it is essential that the Heart Team possesses the necessary knowledge and expertise to determine the most appropriate and individualized treatment strategy for each case. The specific characteristics of patients with primary MR pose challenges in designing large multicenter studies, which are essential to overcome the limitations of the current research protocol and enhance the generalizability of the findings to a broader MR patient population.

## 5. Conclusions

Transapical off-pump heart neochordae implantation is a microinvasive alternative to conventional surgery in symptomatic patients with moderate to severe or severe primary MR. Regarding patients who had favorable MV anatomy for neochords implantation, the procedure has been demonstrated to be safe and to provide good mid-term outcomes for up to 20 [12–39] months in terms of cardiac mortality and freedom from reoperation and rehospitalization.

## Figures and Tables

**Table 1:** Demographic and preoperative data of the patient population.

Parameters	Median [IQR] or n (%)
Age, years	69 [61.75–79.25]
Male, n (%)	29 (69)
BMI (kg/m^2^)	26 [24.50–27.92]
BSA (m^2^)	1.96 [1.82–2.06]
EuroSCORE II (%)	1.79 [1.32–2.48]
STS-PROM MV Repair Score (%)	3.18 [2.28–4.66]
Previous mitral valve surgery, n (%)	1 (2.3)
Know history of CAD, n (%)	18 (42.90)
Dyslipidemia, n (%)	30 (71.40)
Diabetes mellitus type 2, n (%)	8 (19)
Arterial hypertension, n (%)	30 (71.40)
COPD, n (%)	2 (4.80)
AF, n (%)	13 (31)
Smoking, n (%)	16 (38.10)
GFR (mL/min)	70.50 [57.75–83.97]

IQR: InterQuartile range; BMI: body mass index; BSA: body surface area; STS-PROM MV repair Score: Society of Thoracic Surgeons predicted risk of mortality mitral valve repair score; CAD: coronary artery disease; COPD: chronic obstructive pulmonary disease; AF: atrial fibrillation; GFR: glomerular filtration rate.

**Table 2:** Preprocedural mitral valve echo analysis and number of implanted chords.

Parameters	Median [IQR] or n (%)
LAI	1.2 [1.15–1.25]
CI (mm)	4 [2.15–5]
Types of anatomy of the mitral valve	
Type A	35 (83.30)
Type B	4 (9.50)
Type C	2 (4.80)
Type D	1 (2.40)
Number of implanted chords	
2	8 (19)
3	20 (47.6)
4	13 (31)
5	1 (2.40)

IQR: InterQuartile range; LAI: leaflet to annulus index; CI: coaptation index.

## Data Availability

The data presented in this study are available on request from the corresponding author. The data are not publicly available due to patient privacy.

## Abbreviations

MR	Mitral Regurgitation
NYHA	New York Heart Association
MV	Mitral Valve
2D	Two-dimensional
3D	Three-dimensional
TTE	Transthoracic Echocardiography
TEE	Transesophageal Echocardiography
LAI	Leaflet to Annulus Index
CI	Coaptation Index
AML	Anterior Mitral Leaflet
PML	Posterior Mitral Leaflet
AP	Anteroposterior diameter
LVEF	Left Ventricular Ejection Fraction
ESC	European Society of Cardiology
PCI	Percutaneous Coronary Intervention
STS-PROM MV repair score	Society of Thoracic Surgeons Predicted Risk of Mortality Mitral Valve repair score
ePTFE	Expanded polytetrafluoroethylene
